# MERS-CoV pathogenesis and antiviral efficacy of licensed drugs in human monocyte-derived antigen-presenting cells

**DOI:** 10.1371/journal.pone.0194868

**Published:** 2018-03-22

**Authors:** Yu Cong, Brit J. Hart, Robin Gross, Huanying Zhou, Matthew Frieman, Laura Bollinger, Jiro Wada, Lisa E. Hensley, Peter B. Jahrling, Julie Dyall, Michael R. Holbrook

**Affiliations:** 1 Integrated Research Facility, National Institute of Allergy and Infectious Diseases, National Institutes of Health, Ft. Detrick, Frederick, Maryland, United States of America; 2 Department of Microbiology and Immunology, The University of Maryland School of Medicine, Baltimore, MD, United States of America; 3 Emerging Viral Pathogen Section, National Institute of Allergy and Infectious Diseases, National Institutes of Health, Ft. Detrick, Frederick, Maryland, United States of America; Deutsches Primatenzentrum GmbH - Leibniz-Institut fur Primatenforschung, GERMANY

## Abstract

Middle East respiratory syndrome coronavirus (MERS-CoV) presents an emerging threat to public health worldwide by causing severe respiratory disease in humans with high virulence and case fatality rate (about 35%) since 2012. Little is known about the pathogenesis and innate antiviral response in primary human monocyte-derived macrophages (MDMs) and dendritic cells (MDDCs) upon MERS-CoV infection. In this study, we assessed MERS-CoV replication as well as induction of inflammatory cytokines and chemokines in MDMs and immature and mature MDDCs. Immature MDDCs and MDMs were permissive for MERS-CoV infection, while mature MDDCs were not, with stimulation of proinflammatory cytokine and chemokine upregulation in MDMs, but not in MDDCs. To further evaluate the antiviral activity of well-defined drugs in primary antigen presenting cells (APCs), three compounds (chloroquine, chlorpromazine and toremifine), each with broad-spectrum antiviral activity in immortalized cell lines, were evaluated in MDMs and MDDCs to determine their antiviral effect on MERS-CoV infection. While chloroquine was not active in these primary cells, chlorpromazine showed strong anti-MERS-CoV activity, but it was associated with high cytotoxicity narrowing the potential window for drug utilization. Unlike in established cells, toremifene had marginal activity when tested in antigen presenting cells, with high apparent cytotoxicity, also limiting its potential as a therapeutic option. These results demonstrate the value of testing drugs in primary cells, in addition to established cell lines, before initiating preclinical or clinical studies for MERS treatment and the importance of carefully assessing cytotoxicity in drug screen assays. Furthermore, these studies also highlight the role of APCs in stimulating a robust protective immune response to MERS-CoV infection.

## Introduction

Middle East respiratory syndrome coronavirus (MERS-CoV) was first isolated in Saudi Arabia in 2012 from a patient with severe acute respiratory disease complicated by renal failure [1, 2]. Since that time, the virus has caused sporadic outbreaks of mild-to-severe respiratory disease. Approximately 80% of human cases have been reported in Saudi Arabia with 211 cases occurring in the first 9 months of 2017 [3]. Beginning in May 2015, a large hospital-associated outbreak of MERS occurred in the Republic of Korea. The outbreak in Korea resulted in a total of 186 MERS-CoV cases, including 36 deaths, and was the largest outbreak of MERS occurring outside of the Arabian Peninsula [4]. This outbreak highlighted the risk of international dissemination of MERS-CoV and the continued risk of nosocomial infection. As of September 6, 2017, the number of confirmed global cases of MERS-CoV infection reported to World Health Organization was 2079 cases in 27 countries with 722 fatalities, resulting in a case fatality rate around 35%[3].

MERS-CoV is a zoonotic virus that is transmitted from animals to humans with camels likely serving as the principal host for MERS-CoV [5]. While nosocomial infections are common, barrier nursing practices can limit spread of the virus as the virus does not seem to pass easily from person-to-person unless close contact occurs [6]. In humans, MERS-CoV infection typically causes a lower respiratory tract disease such as pneumonia, and common symptoms include fever, cough, sore throat, myalgia, and shortness of breath [7]. Symptoms such as gastrointestinal complications and renal failure have also been reported in patients, especially those with severe chronic illness such as diabetes [6, 8]. Systemic dissemination has been documented in locations such as the circulatory system and respiratory tract [9].

In the studies presented here, we had two principal objectives. The first was to determine whether human antigen presenting cells (APCs) were permissive to MERS-CoV infection. The second objective was to determine if these cells were suitable or appropriate for secondary screens for drugs that have been identified as effective in continuous culture cell lines. Macrophages and dendritic cells (DCs) are professional APCs linking innate and adaptive immunity. These and other APCs act as a first defense against viral infection by stimulating immune surveillance, priming, and tolerance [10, 11]. Appropriately functioning APCs are critical for the ability to mitigate infection and limit the development of disease. APCs are abundant in the respiratory tract where they provide immune surveillance and respond to local tissue inflammation in the airways and the distal lung. An important role of APCs is mitigating infection by producing cytokines that stimulate an inflammatory response and recruiting memory and effector cells to the site of infection [12]. Professional APCs are also an important source of type I interferons (IFN-α/β). Type I IFNs have a significant bystander effect on uninfected neighboring cells by inducing an antiviral state, activating innate immune cells, and priming adaptive immunity.

Currently, no prophylactic or therapeutic options are proven as effective interventions for infection with MERS-CoV, severe acute respiratory syndrome coronavirus (SARS-CoV), or any other coronaviruses. To rapidly identify potential therapeutic options against emerging viral infections, investigators have adopted the approach of screening existing licensed drugs for efficacy against novel viral pathogens. Screening licensed drugs could expedite the implementation of new medical countermeasures by providing an avenue for off-label use of compounds shown to be useful for the treatment of specific viral diseases.

A number of pharmaceutical agents have potential for the treatment of coronaviruses, including neurotransmitter inhibitors, estrogen receptor antagonists, kinase signaling inhibitors, protein-processing inhibitors, and antiparasitic agents [13, 14]. Results from previous studies found toremifene citrate (TOMF), chlorpromazine (CPZ) and chloroquine (CQ) to be effective in blocking MERS-CoV and SARS-CoV infection *in vitro* in established cell lines such as Vero E6 and Huh 7 cells [13, 14]. We established a cell differentiation method to generate large amounts of monocyte-derived macrophages (MDMs) and dendritic cells (MDDCs) of high quality (>95% purity) for evaluating compounds against other viruses such as yellow fever and Ebola viruses [15]. To address pharmaceutical efficacy against MERS-CoV infection in primary cells critical for blocking infection, we tested several candidate MERS-antivirals in human MDMs and immature dendritic cells. The tested compounds included TOMF, an estrogen receptor antagonist, CPZ, a neurotransmitter inhibitor, and CQ, an antimalarial agent.

## Materials and methods

### Biosafety

All viral infection-related work was performed in the biocontainment laboratory at the Integrated Research Facility (IRF) of National Institute of Allergy and Infectious Disease (NIAID)/ National Institute of Health (NIH) using procedures and processes appropriate for the facility and the agent under study. All staff performing this work were trained to work in the biocontainment space and with MERS-CoV.

### Cells, compounds, and virus

Vero E6 cells (ATCC 1568) were obtained from American Type Culture Collection (ATCC, Manassas, VA) and maintained in Dulbecco’s modified Eagle’s medium with 10% heat inactive fetal bovine serum (FBS) following manufacture’s instruction.

Whole blood from which APCs were isolated was acquired from either the National Cancer Institute (Frederick, MD) or the Biological Specialty Corporation (Colmar, PA).

Chlorpromazine hydrochloride (CAS 69-09-0) and chloroquine diphosphate salt (CAS 50-63-5) were purchased from Millipore Sigma, St. Louis, MO. Toremifene citrate (CAS 89778-27-8) was purchased from Sequoia Research Products, Pangbourne, United Kingdom. Dimethyl sulfoxide (DMSO) (Millipore Sigma, St. Louis, MO) was used as a solvent for the high-throughput screening assay described below.

MERS-CoV Jordan strain (Hu/Jordan-N3/2012, GenBank accession no. KC776174.1;[2]) was kindly provided by Drs. Kanta Subbarao (NIH, Bethesda, MD, USA) and Gabriel Defang (Naval Medical Research Unit 3, Cairo, Egypt). The virus was amplified by infection of Vero E6 cells at a multiplicity of infection (MOI) of 0.01. The infected cells were monitored under a microscope daily. Once cytopathic effects were visible on day 4 post-inoculation, virus-containing supernatants were harvested, clarified by centrifugation, and aliquoted for storage at -80°C. The MERS-CoV stock titer was determined by plaque assay on Vero E6 cells (see below).

### Primary cell generation and immunophenotyping by flow cytometry

Human MDMs and MDDCs were differentiated at the IRF as described previously [15]. Briefly, CD14^+^ monocytes were isolated from human peripheral blood mononuclear cells for differentiation into MDMs and MDDCs. MDMs were differentiated by culturing the purified CD14+ monocytes with human macrophage-colony stimulating factor (M-CSF) (R&D systems, Minneapolis, MN) and KPB-M15 conditioned medium (SCGF Research Laboratory, Ukyo-ku, JP) for 7 days. To generate MDDCs, CD14+ monocytes were supplied with 20 ng/ml of human granulocyte macrophage-colony stimulating factor (GM-CSF) and 10 ng/ml of interleukin (IL)-4 in R-10 medium [RPMI-1640 (Lonza, Allendale, NJ)] containing 10% heat inactivated FBS (Millipore Sigma). The cells were then incubated for 6 days at 37°C and 5% CO_2_ with a change of medium every second day. Immature MDDCs were harvested at this point or stimulated to induce maturation. To induce maturation, the medium was replaced with a MDDC maturation cocktail (R-10 medium containing 10 ng/ml of tumor necrosis factor (TNF)-α, 10 ng/ml of IL-1β, 15 ng/ml of IL-6, 1 μg/ml of prostaglandin E2 (PEG2), 20 ng/ml of GM-CSF, 10 ng/ml of IL-4) on day 6 post-plating and incubated overnight at 37°C and 5% CO_2_. The cells were then collected for phenotyping and further assays.

**Fig 1:**
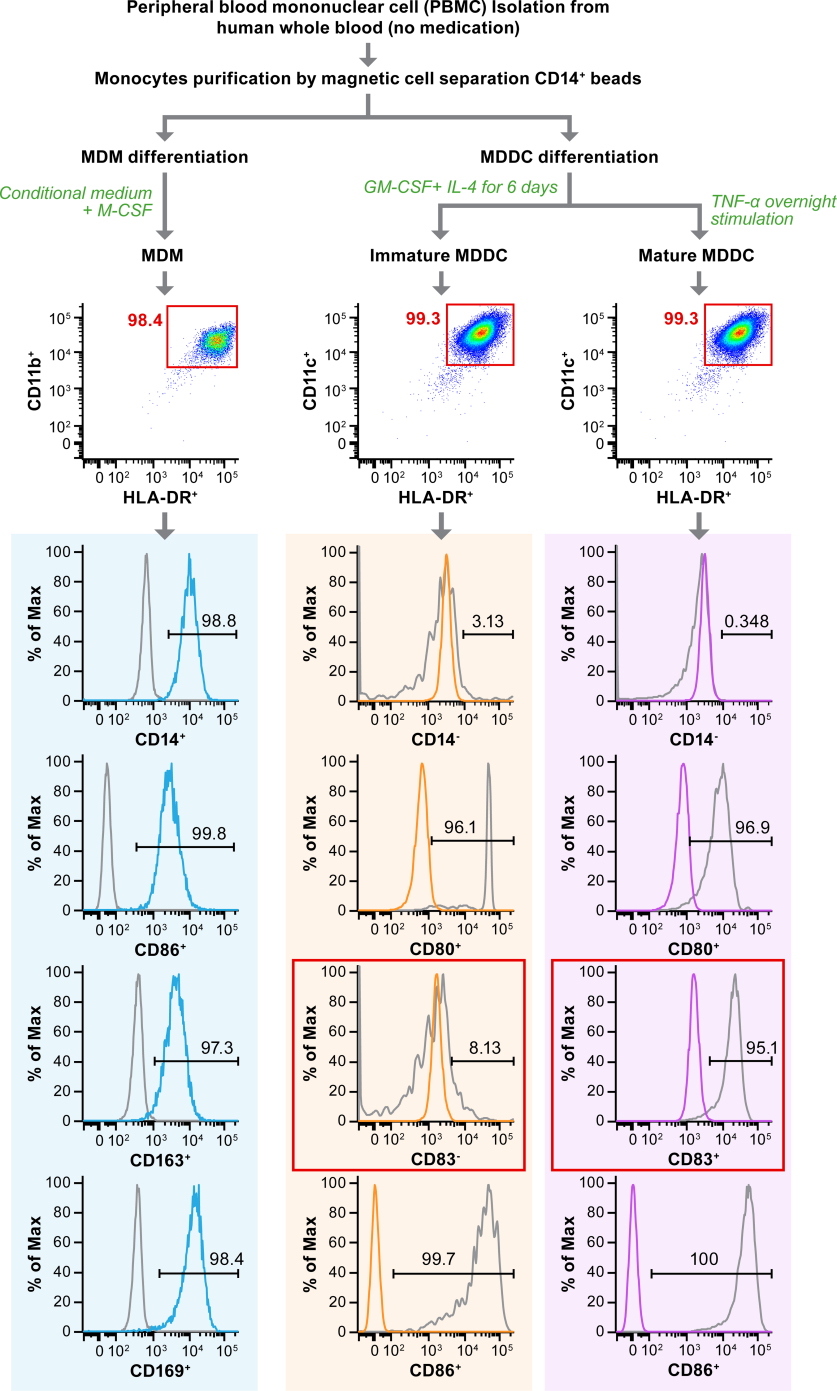
Differentiation of macrophages and dendritic cells from peripheral blood mononuclear cells. CD14+ monocytes were isolated from human peripheral blood mononuclear cells (PBMCs). Cells were then differentiated to monocyte-derived macrophages (MDMs) by adding macrophage-colony stimulating factor (M-CSF) or to immature monocyte-derived dendritic cells (MDDCs) by adding granulocyte macrophage-colony stimulating factor (GM-CSF) and interleukin-4 (IL-4). Immature MDDCs were exposed to MDDC maturation cocktail (R-10 medium containing 10 ng/ml of tumor necrosis factor (TNF)-α, 10 ng/ml of IL-1β, 15 ng/ml of IL-6, 1 μg/ml of prostaglandin E2 (PEG2), 20 ng/ml of GM-CSF, 10 ng/ml of IL-4) overnight for maturation. MDMs and MDDCs were characterized by flow cytometry for expression of major cell-surface markers. Using these protocols, the MDMs are predominantly CD14+, CD11b+, HLA-DR+, CD169+, CD163+, and CD86+. The MDDCs are predominantly CD14-, CD11c+, HLA-DR+, CD80+, and CD86+. Mature MDDCs are CD83+, while immature MDDCs are CD83-.

MDMs and MDDCs were stained and analyzed as mentioned previously [15]. The MDM antibody panel contained anti-human monoclonal antibodies specific for CD14 (fluorescein), HLA-DR (V500), CD11b (PB), CD163 (APC), CD169 (PE), CD86 (PE-CY7) (BD Biosciences). The panel for testing MDDCs included CD11c (pacific blue), HLA DR (V500), CD14 (phycoerythrin [PE]), CD40 (fluorescein), CD80 (quantum dot 605), CD83 (Peridinin chlorophyll protein cyanine5.5), CD86 (PE-cyanine7). Viability was determined by using LIVE/DEAD^®^ Fixable Yellow Dead Cell Stain (Thermo Fisher Scientific, Waltham, MA). Analysis of the MDM and DC phenotype was completed by flow cytometric analysis on a BD Fortessa LSR II cytometer (BD Biosciences, San Jose, CA, USA). Results were analyzed using Flowjo software (FLOWJO, Ashland, OR), and cells were gated around the live, singlet monocyte population. Additional cell surface marker expression was determined based on the double-positive population using the gating strategy described in Fig 1.

### MERS-CoV growth kinetics on MDMs and MDDCs

MDMs and MDDCs (immature and mature) were seeded at 5 *×* 10^5^ cells per well in 48-well plates and incubated overnight at 37°C and 5% CO_2_. The next day, the cells were infected at a MOI of 2 for 1 (hour) h at 37°C and 5% CO_2_. After the inoculum was removed, cells were washed twice with phosphate-buffered saline and cultured with R-10 medium. Cell-free supernatants were collected, aliquoted to multiple microtubes at the indicated time-points, and frozen at -80°C until used.

### MERS-CoV titration by plaque assay

The titer of virus stocks and cell-free supernatants collected from kinetics studies were determined by plaque assay on Vero E6 cells. Test samples were diluted serially 1:10, and 100 μl of the sample was added to each well of 6-well plates containing an 80–90% confluent monolayer of Vero E6 cells. The plates were then incubated for 1 h at 37°C and 5% CO_2_ with rocking every 15 mins for virus adsorption and infection. Following incubation, a pre-warmed semi-solid overlay (0.8% tragacanth [f/c] (Millipore Sigma) mixed in 1x Eagle’s minimum essential medium containing 2% FBS) was gently added to the cells. The plates were then incubated for 5 days at 37°C and 5% CO_2_ without being disturbed. To fix the plates, the overlay was aspirated, and the cells were fixed and stained with neutral buffered formalin (NBF) (Thermo Fisher Scientific) containing 0.2% crystal violet (f/c) (Millipore Sigma) for 30 min at room temperature. The plates were then washed with running water and air-dried. The plaques were then enumerated, and the virus titer was calculated.

### Multiplex assay for detection of cytokines and chemokines in MERS-CoV-infected MDMs and MDDCs

Inflammatory mediator expression was measured using a custom human 10-plex cytokine bead arrays (Cat# HCYTOMAG-60K, Millipore Sigma, Billerica, MA) on a Luminex FLEXMAP 3D^TM^ system equipped with xPonent 4.2 software. (Luminex, Austin, TX). Cytokines and chemokines included in the panel were: IFN-α2, IFN-γ, TNF-α, IL-6, IL-8, IL-12p40, monocyte chemotactic protein (MCP)-1, macrophage inflammatory protein (MIP)-1α, interferon-inducible protein (IP)-10, and regulated upon activation normal T-cell expressed and secreted (RANTES). The assays were performed according to the manufacturers' instructions in 96-well Mylar plates (Millipore) as described previously [15]. Cell-free supernatants harvested from kinetics studies were mixed with beads coated with capture antibodies to various cytokines and chemokines. The mixtures were incubated with biotinylated detection antibodies. Finally, PE-conjugated streptavidin was added, and the fluorescent signals were measured using a FLEXMAP equipped with xPonent 4.2 software (Luminex Corp).

The raw data were measured as mean fluorescence intensity (MFI), and the concentration of each analyte was calculated using a 4- or 5-parameter logistic fit curve generated for each analyte from the 6-point concentration standard curve. The concentrations of all the analytes in the quality control reagents were found to be within the expected ranges. The lower limit of quantification (LLOQ) was determined using the lowest point on the standard fit curve that was at least 3 times above background. The calculation of the LLOQ was performed by subtracting the MFI of the background (diluent) from the MFI of the lowest standard concentration and back-calculating the concentration from the standard curve. Data were further analyzed using Prism 6.0 software (GraphPad Software, La Jolla, CA).

### Antiviral activity against MERS-CoV determined by cell-based assay

MDMs or MDDCs (100 μl) were plated in black opaque (cytotoxicity assay) or clear bottom (testing viral inhibition) 96-well Greiner microplates (Greiner Bio-One, Monroe, NC, 655948) at 1 × 10^5^ cells/well and incubated at 37°C and 5% CO_2_ overnight. Drugs in powder formulations were dissolved in (DMSO) (Millipore Sigma). Final DMSO concentrations were lower than 0.05%. Drug solutions diluted in RPMI-1640 with 10% FBS (R-10 medium) to a 4X concentration were added into a 96-well, cell-free, drug dilution plate. Drugs (50 μl/well) were transferred from the drug dilution plates to cell plates using the 96-well liquidator (Rainin Instrument, Oakland, CA) 1 h prior to infection. Duplicate cell plates for each cell type were infected with MERS-CoV at a MOI of 0.1 by adding 50 μl of virus diluted in R-10 medium directly to the drug mixture in the cell plate and the plates incubated at 37°C and 5% CO_2_. After 48 h, cell culture supernatants were collected, and the cells were fixed with 10% NBF. The collected supernatants were clarified by centrifugation and stored at -80°C for future use.

To quantify the antiviral activity of the candidate compounds, a cell based immunofluorescence enzyme-linked immunosorbent assay was used as described previously [13]. The fixed plates, as described above, were stained with a rabbit polyclonal antibody to the MERS-CoV-EMC/2012 S protein (Sino Biological) at 1:1000 followed by staining with Alexa Fluor 594-conjugated goat anti-Rabbit IgG (H+L) antibody (Thermo Fisher Scientific) at 1:2500. Fluorescence was quantified on a plate reader (Infinite M1000 Pro; Tecan US, Morrisville, NC) with an excitation wavelength of 590 nm and an emission wavelength of 617 nm.

Cytotoxicity assays were performed in parallel to measure drug toxicity. One black opaque cell plate for each cell type was mock infected using R-10 medium under the same conditions as the infected cells, and cell viability was measured using the CellTiter Glo Luminescent Cell Viability Assay kit (Promega, Madison, WI) according to the manufacturer’s instructions. Luminescence was read on the Infinite® M1000 Pro plate reader (Tecan).

### Quantification of antiviral activity tested by TCID_50_

Antiviral activity was evaluated by means of a 50% tissue culture infectious dose (TCID_50_) assay using the Spearman/Kaerber method [16, 17]. Briefly, a 10-fold serial dilution, 10^−1^ to 10^−7^, was made from cell-free supernatant samples collected from the drug efficacy evaluation studies for cell-based enzyme-linked immunosorbent assay. These dilutions were used to infect Vero E6 cells on 96-well plates with seven infected wells for each dilution. Samples containing only cell culture media were used as a negative control, and virus stock was used as a positive control. Test samples were added to each well at a volume of 100 μl. The plates were then incubated for 6 days at 37°C and 5% CO_2_ to observe the MERS-CoV-induced cytopathology. Following the incubation, the media were removed, and the cells were fixed and stained with 10% NBF containing 0.2% crystal violet. The plates were incubated for 30 min at room temperature and then washed with running water. The TCID_50_ titer was determined by identifying the 50% endpoint in cytopathic effects in the cell monolayer. The results were calculated by applying the Spearman/Kaerber method and presented as log_10_ TCID_50_/ml.

### Statistical data analysis

Non-linear regression analysis was performed to calculate 50% effective concentration (EC_50_) of compounds and their specific toxicity (50% cytotoxicity concentration, CC_50_s) using Prism 6.0 software (GraphPad Software) from the dose-response curves of the drugs. Selectivity index (SI) is defined as the ratio of the concentration of 50% cellular cytotoxicity to 50% effective concentration (SI = = CC_50_÷EC_50_), indicating the relative efficacy of a compound in viral replication inhibition as opposed to causing cytopathic effect. Error bars represent the standard deviation of three replicates unless indicated otherwise. Statistical significance determinations were performed using the results of at least two individual experiments or duplicate or triplicate replicates from a single experiment.

## Results

### Immunocharacterization of MDMs and MDDCs by flow cytometry

For these studies, MDMs and MDDCs were carefully characterized to ensure uniformity and reproducibility between experiments. Differentiated MDMs and MDDCs were characterized by flow cytometry as performed previously [15]. The MDMs generated were predominantly CD14^+^, CD11b^+^, HLA-DR^+^, CD169^+^, CD163^+^, and CD86^+^, confirming that the MDM population was highly purified, and over 95% of the cells generated were positive for macrophages (Fig 1). Phenotyping of human MDDCs showed they were CD14^-^, CD11c^+^, HLA-DR^+^, CD40^+^, CD80^+^, and CD86^+^. The DC maturation marker, CD83, was present on 95.1% of mature MDDCs (Fig 1) compared to 8.13% of the immature MDDCs and 0.98% of the unstained control cells (Fig 1). The isolation protocol used in these studies consistently generated highly purified phenotypically homogeneous APCs with minimal inter-experimental or inter-operator variability.

### MERS-CoV propagation in human MDMs and MDDCs

**Fig 2:**
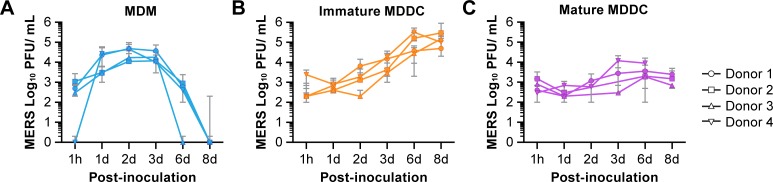
Viral titers from supernatants of MERS-CoV-infected macrophages and dendritic cells via plaque assay. (A) Macrophages (MDMs), (B) immature monocyte-derived dendritic cells (MDDCs), and (C) mature MDDCs were infected at an MOI = 2 with MERS-CoV Jordan variant. Culture supernatants were collected at indicated times post-inoculation to detect viral titers. Each line represents cells from each individual donor. Titrations were performed in triplicate with data points representing the mean of the triplicate values.

To determine if primary APCs are permissive to MERS-CoV infection, kinetics studies were performed to measure virus titer following infection using cells isolated from four different donors. A 2- to 4-log_10_ increase in viral titers was observed in MDMs and immature MDDCs despite variation between donors (Fig 2A and 2B). No significant increase in viral titer was observed in mature MDDCs up to 8 days pi (Fig 2C) indicating a restriction in virus infection or virus replication. Virus clearance in MDMs began at day 3 pi, and no virus could be detected by 6 to 8 days pi. However, MERS-CoV continued to propagate in immature MDDCs up to 8 days pi, demonstrating differential infection and replication capabilities in MDMs and immature MDDCs.

### Cytokine and chemokine production upon MERS-CoV infection

**Fig 3:**
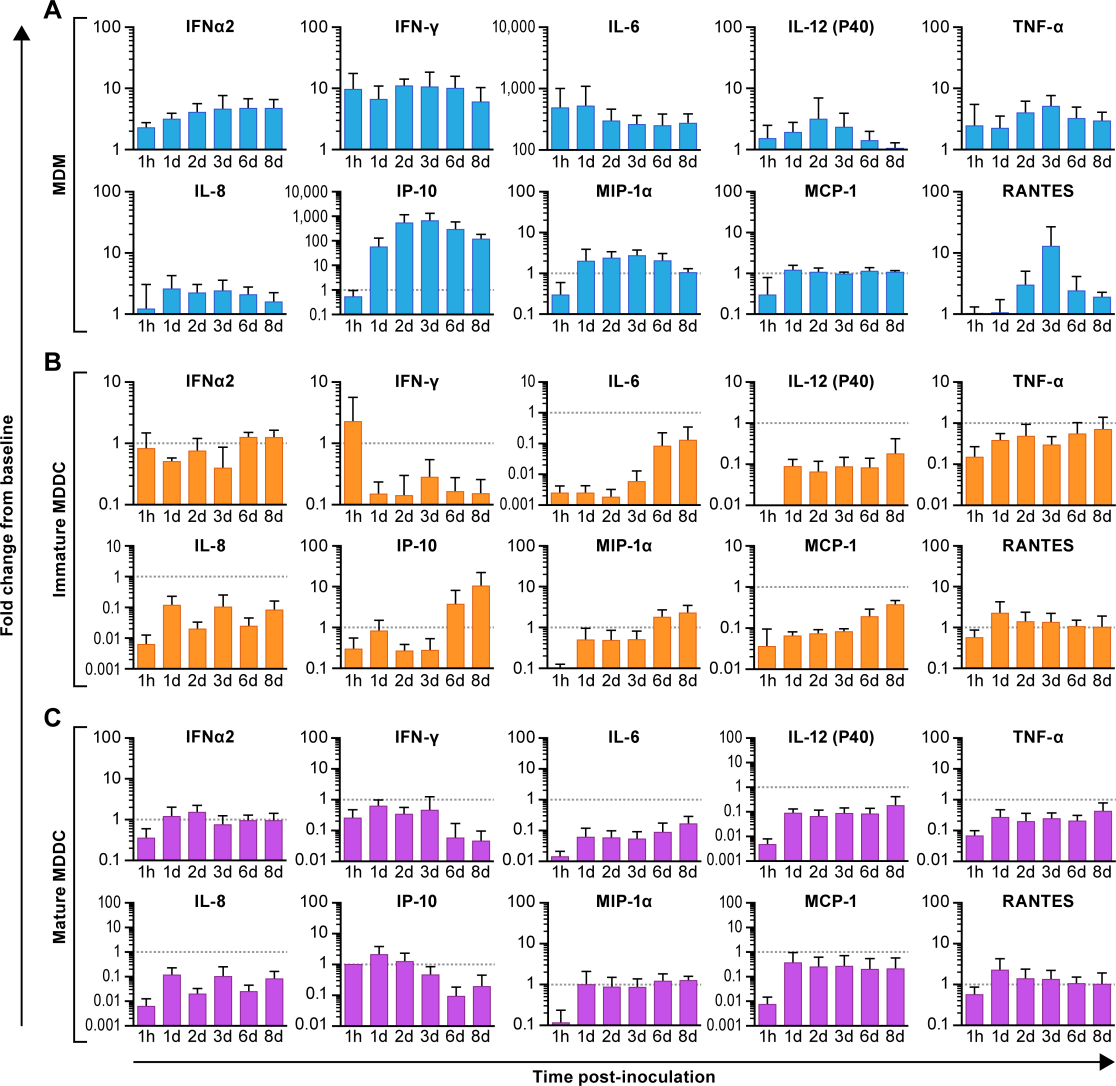
Cytokine and chemokine responses to MERS-CoV infection from certain innate immune primary cells. (A) Human macrophages (MDMs) (B), immature monocyte-derived (MDDCs), and (C) mature MDDCs were inoculated with MERS-CoV Jordan variant at an MOI = 2 or subjected to mock infection. From cell culture supernatants, the fold change in immunoregulatory cytokines and chemokines relative to that of mock-infected cells incubated for the same period of time, were determined by multiplex assay. All data points represent triplicate biological replicates from four donors.

To compare the ability of MERS-CoV to induce innate immune responses in three types of APCs, the release of cytokines and chemokines was measured from virus- or mock-infected cells. The 10-plex human panel included the antiviral cytokines (IFN-α2 and IFN-γ), proinflammatory cytokines (TNF-α, IL-6, and IL-12p40), and chemokines (IP-10, MCP-1, MIP-1α, RANTES and IL-8). In MDMs, MERS-CoV infection resulted in increased concentrations of almost all antiviral or proinflammatory cytokines and most chemokines (except MCP-1), many of these cytokines or chemokines were released as soon as 1 h pi (Fig 3A) and may have been the result of direct cell stimulation rather than active infection. The high induction of IFN-γ, and IP-10 over the course of the infection in MDMs was dramatic, indicating a very strong antiviral response was induced upon MERS-CoV infection. MERS-CoV infection repressed IL-6 expression from MDM and this repression decreased over the course of several days (Fig 3A) with a similar response profile seen with chemokines MIP-1α and MCP-1. Increased MIP-1α and IP-10 expression was observed in immature MDDCs (Fig 3B). However, other than a bolus of IFN-γ released immediately after the infection of MDDC, the release of most of the cytokines or chemokines from immature and mature MDDCs were negligible over the course of the MERS-CoV infection relative to mock-infected cells (Fig 3B and 3C). As expected in a human population, considerable donor-to-donor variation in the cytokine response was observed following infection with MERS-CoV.

### Effect of chloroquine and chlorpromazine on MERS-CoV replication in MDMs and immature MDDCs

**Fig 4:**
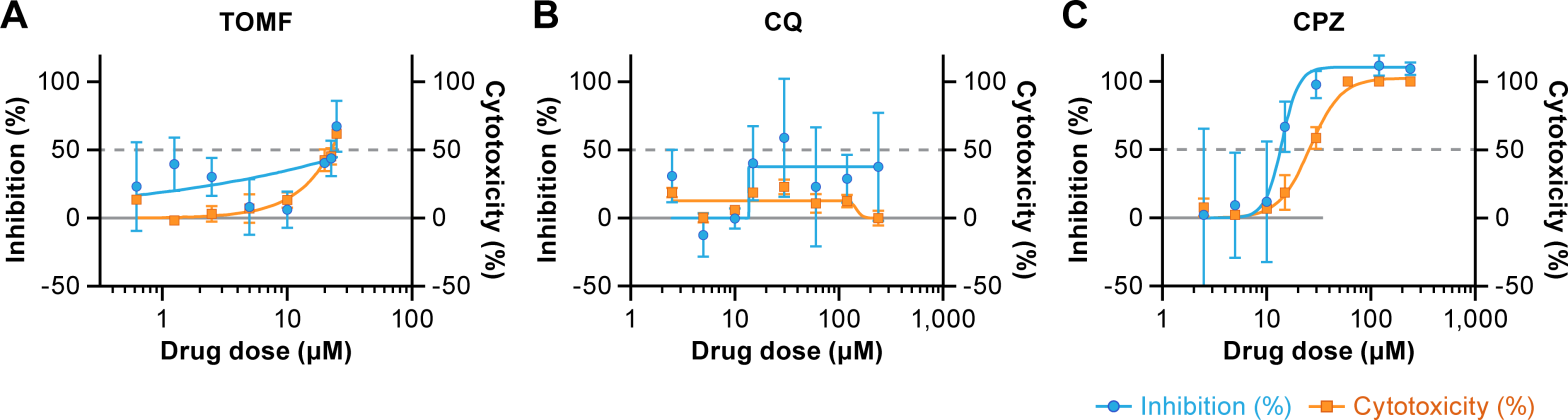
Antiviral activity of three compounds on MERS-CoV infection in MDMs. (A) Effect of pretreatment of monocyte-derived macrophages (MDMs) with toremifene (TOMF), (B) chloroquine (CQ), and (C) chlorpromazine (CPZ) prior to MERS-CoV exposure on subsequent infection. The effect of these compounds on the cytotoxicity of macrophages without virus was also studied. MERS-CoV S protein was detected in infected cells using an immunofluorescence assay. The antigen was detected with a rabbit polyclonal antibody to S protein followed by staining with Alexa Fluor 594-conjugated goat anti-rabbit antibody. Percent inhibition of MERS-CoV infection is shown in blue, and percent cytotoxicity of the compounds without virus is shown in orange. Dotted gray line indicates half maximal effective concentrations (EC_50_) and 50% cytotoxicity concentrations (CC_50_). The selectivity index (SI) is defined as CC_50_÷EC_50_. Results are representative of 2 individual experiments with 3 replicates in each experiment (means ± standard error of the means [SEM]).

A cell-based immunofluorescence assay was applied to evaluate the antiviral effectiveness of CQ, CPZ, and TOMF in MDMs, which adhere to 96-well plates and could be fixed and stained. Cytotoxicity assays were performed in parallel using mock-infected cells after drug addition. TOMF performs well inhibiting MERS-CoV replication in Vero E6 cells (EC_50_ = 12. 9 μM) [13]. In MDMs, the activity of TOMF at the dose range tested was too low to determine an EC_50_ and the cytotoxicity was relative high (Fig 4A). Unlike in Vero E6 cells (EC_50_ = 6.3 μM) [13], CQ did not show any detectable antiviral activity in MDMs, although no cytotoxicity was associated with this compound (Fig 4B). CPZ inhibited MERS-CoV in Vero E6 cells (EC_50_ = 9.5 μM) with low cytotoxicity [13]. However, in MDMs, the cytotoxicity of CPZ was relatively high (Fig 4C, CC_50_ = 25.64 μM. Antiviral activity of CPZ (EC_50_ = 13.58 μM) did not separate well from cytotoxicity and led to a low selectivity index (SI = 1.9).

**Fig 5:**
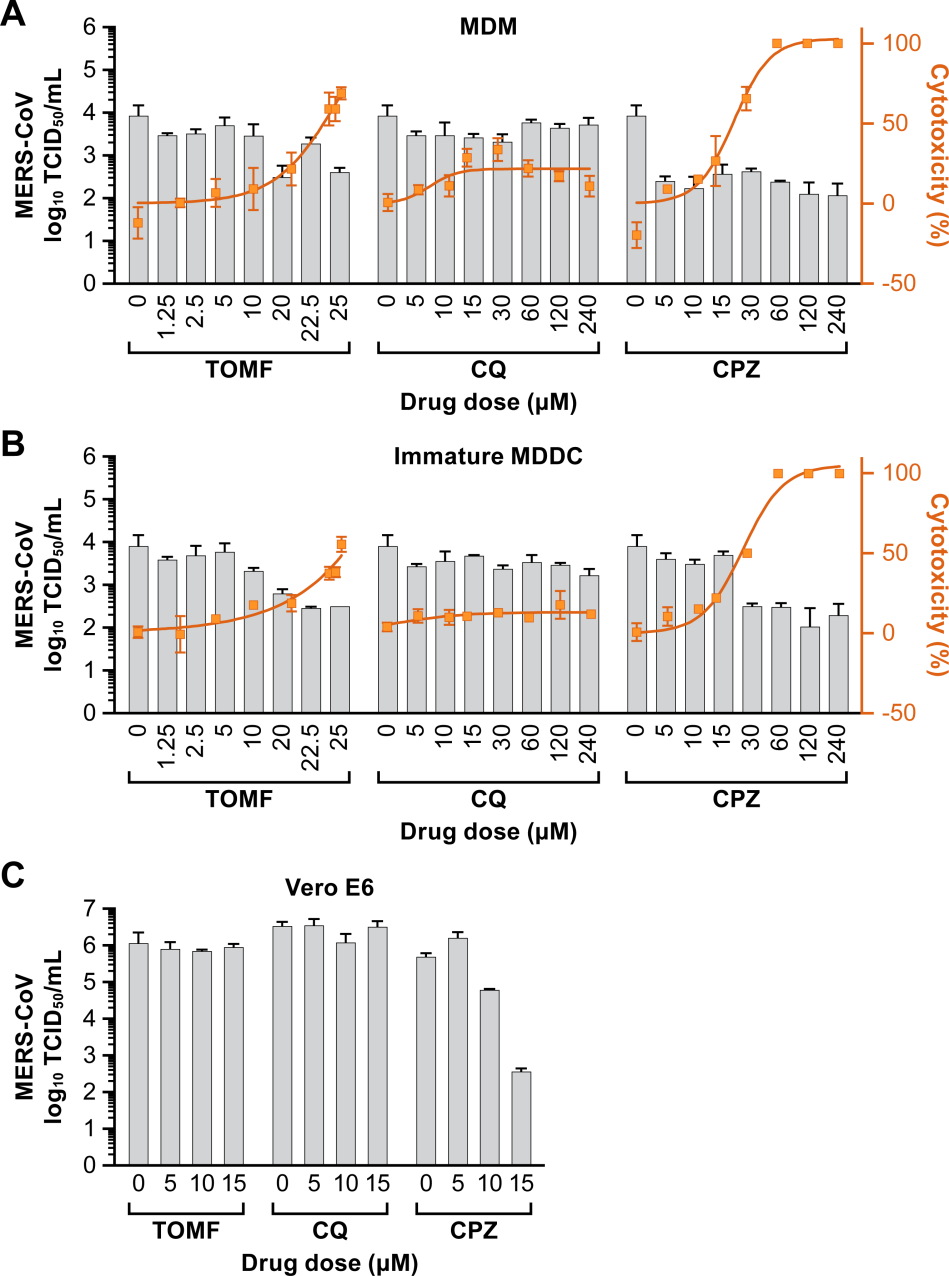
Antiviral activity of three compounds on MERS-CoV infection in MDMs and immature MDDCs. (A) Monocyte-derived macrophages (MDMs), (B) immature dendritic cells (MDDCs) and (C) Vero E6 cells were pretreated with toremifene (TOMF), chloroquine (CQ), and chlorpromazine (CPZ) for 1 h followed by inoculation with MERS-CoV Jordan variant at an MOI = 0.1. Supernatants collected at 72 h post infection (hpi) for APCs and 48 hpi for Vero E6 cells were inoculated onto Vero E6 cells pre-seeded overnight in 96 well plates and incubated for 6 days. Wells with cytopathic effects were counted, and TCID_50_ was determined using the Reed-Muench method. Cytotoxicity was measured in parallel using luminescent cell viability assay at 48 h after compounds addition to the mock-infected MDMs and immature MDDCs. Antiviral activity is shown in gray and cytotoxicity is shown in orange. Results are representative of 2 individual experiments with 3 replicates in each (means ± standard error of the means [SEM]).

The cell-based immunofluorescence assay could not be applied to immature MDDCs due to their poor adherence to the 96-well plate; cells could not be fixed for further antibody staining. Therefore, the efficacy of the candidate compounds on MERS-CoV-infected APCs was evaluated by a median TCID_50_ assay. Mature MDDCs did not produce detectable infectious virus after infection and were therefore excluded (Fig 2C) in these drug activity studies. Cell culture supernatants saved from the cell-based assay were titrated by TCID_50_ to detect MERS-CoV to evaluate the efficacy of the compounds. There was no virus yield reduction in CQ-treated MDMs or immature MDDCs, though the cytotoxicity was relativity low (Fig 5A and 5B). In CPZ treated cells, around a 2 log_10_ virus reduction was observed in both cell types. However, high cytotoxicity narrows the therapeutic window in both MDMs and MDDCs (Fig 5A and 5B). A 1–1.5 log_10_ virus reduction was measured in TOMF-treated cells, but only when the dose is higher than 20 μM, with a corresponding increase in cytotoxicity (Fig 5A and 5B). These observations were similar when compared to the cell-based assay mentioned above. Virus yield reduction data for TOMF, CQ and CPZ in Vero E6 cells showed that CPZ was active against MERS-CoV infection with a 3.1 log_10_ drop at the drug dosage of 15 μM, but TOMF and CQ were not active (Fig 5C). These data suggest a strong correlation between VeroE6 cells and MDMs when virus titers are determined by TCID_50_.

## Discussion

As sporadic outbreaks of MERS continue to occur, our current options for treatment of life-threatening zoonotic coronavirus infections in humans are still lacking. The lack of treatment is due to the poorly understood pathogenesis of MERS and other coronavirus infections, despite the extensive research efforts triggered by the 2003 SARS outbreak [18].

Human APC cells, MDMs and MDDCs, play important roles in bridging innate and adaptive immunity during viral infections by presenting viral antigens to T cells and by secreting appropriate cytokines and chemokines [19]. MDMs and immature MDDCs reside at the sites where most infections occur, such as the mucosal surfaces within the respiratory tract. In their role in the immune response, APCs are also a potential initial site for viral replication. Additionally, APCs can act as viral reservoirs for further dissemination to organs as already shown for other human infectious diseases like measles and human immunodeficiency virus (HIV) infection [20, 21].

In this study, we assessed the characteristics of MERS-CoV growth kinetics in MDMs and MDDCs. We found that MERS-CoV can readily infect and robustly replicate in human MDMs and immature MDDCs, while the virus does not establish a notable infection in mature MDDCs. High viral infectivity in MDMs and immature MDDCs indicates that the virus can overcome the initial antiviral response, but that the maturation of MDDC imparts a restriction that prevents virus replication.

The capacity of MDDC to stimulate T cells is closely related to their maturation stage [22]. Immature DCs are highly specialized at antigen uptake and processing, but are poor stimulators of primary immune responses [23–25]. Productive infection of immature DCs may delay the activation of T cells, therefore allowing more time for MERS-CoV replication or dissemination. An additional reason that immature MDDCs may be more susceptible to MERS-CoV infection than mature MDDCs to MERS-CoV could be their ability to internalize and process viral antigens. After uptake of antigens or pathogens, immature MDDCs may undergo maturation, migrate to lymphatic tissues, and trigger adaptive immune responses. In theory, the ability of sampling and presenting antigen should be enhanced through MDDCs maturation. For example, immature MDDCs may promote viral dissemination of SARS-CoV within the host by recruiting the virus to enter and damage the lymphatic tissues [26].

A recent study has shown that abortive herpes herpesvirus (HHV)-1 (herpes simplex virus-1) infection of mature MDDC is able to modulate their inhibition of T-cell stimulatory function [27]. Endocytosis in mature DCs is slowed somewhat relative to immature DCs, potentially the result of changes to their endocytic mechanism or receptor expression [28]. Similarly, virus was disseminated systemically in a patient with MERS [29].

Following MERS-CoV infection, the induced immune response in APCs was consistent with their susceptibility to infection and the kinetics of virus propagation. The production of proinflammatory cytokines is essential for host resistance against MERS-CoV infection. Infection of MDMs with MERS-CoV appreciably induced the secretion of antiviral cytokines (IFN-α2, IFN-γ, and IL-12p40), moderately or appreciably up-regulated proinflammatory cytokines (TNF-α, IL-6), and variably upregulated inflammatory chemokines (MIP-1α, IP-10, RANTES, IL-8). Clinical studies have shown that expression of proinflammatory cytokines/chemokines, such as IL-6, IL-8, IFN-γ, or IP-10, is highly correlated with disease severity and mortality of SARS patients [30]. Nicholls *et al* reported that macrophages in lung tissues of SARS patients were identified as the location where the “cytokine storm” and virus pathogenesis originated [31]. MDMs secrete proinflammatory cytokines and chemokines to activate antiviral mechanisms, which may cause the dysregulated production of these inflammatory mediators leading to damage to neighboring tissues [32]. Expression of IL-6 and TNF-α are a potential indicators of severe respiratory viral infection, such as SARS and human infection by avian influenza viruses [33]. Our data indicate the possibility of an IFN- γ Th1-mediated inflammatory response that may cause severe MERS disease before an adaptive host immune response is mounted. However, restriction of virus growth in mature MDDCs results in no antiviral activation by IFN-α2. Nevertheless, only moderate expression of IP-10 and minimal induction of MIP-1α, MCP-1, and RANTES were observed in immature MDDCs. Significant upregulation of most cytokines and chemokines was not observed in either mature or immature MDDCs. Overall, the limited antiviral cytokine response in MDDCs suggests a possible mechanism of immune escape during MERS-CoV infection as seen in SARS-CoV infection [26]. A review of the literature suggests that there is a significant gap in knowledge regarding the role on intracellular regulatory mechanisms such as pathogen recognition receptors (PRR) in the control of coronavirus infection. In this study there was essentially no change in the concentration of cytokines/chemokines measured in mature (and non-permissive) MDDCs. In immature MDDCs, while the measured concentration was less than the control cells in many cases, the concentrations of a number of cytokines/chemokines (IL-6, IP-10, MIP-1α, MCP-1) increased over the course of the infection, specifically on days 6 and 8 when titers were highest. These data suggest that PRR may have been activated, as would be anticipated in a productive viral infection, but the scale and scope was not evaluated. In addition, data obtained for cytokine release very early after infection cannot be properly evaluated due to the potential presence of cellular components in the virus preparations.

In several reports of immune responses triggered by APC, findings are similar, or conflicting compared to our results. For example, Tynell, *et al*. stated that MERS-CoV replicates poorly in human macrophages and monocyte-derived dendritic cells, but stimulates an immune response [34]. Scheuplein, *et al*. reported that human macrophages and MDDCs do not mount an immune response following MERS-CoV infection [35]. However, Zhou, *et al*. found MERS-CoV can infect and robustly replicate in MDMs and immature MDDCs triggering the aberrant production of proinflammatory cytokine/chemokine responses [19, 32]. These discrepancies may relate to differences in experimental conditions, the protocols for generating and phenotyping MDMs and MDDCs in different laboratories, or variability of individual blood donors [36].

Appreciable efforts have been made to identify novel antiviral therapeutics for MERS-CoV since the initial outbreak occurred. Compounds that target host effectors could be beneficial during the course of infection [37]. MERS-CoV drug screens have been performed mostly in established cell lines such as Vero and Huh 7 cells. Very little drug evaluation has been performed in primary cell lines. Here we picked three FDA-approved broad-spectrum inhibitors (CPZ, CQ, TOMF) that were shown to be effective against MERS-CoV infection in immortalized cell lines [13, 14] and evaluated their antiviral activities in MDMs and MDDCs. We excluded mature MDDCs for drug screening due to the lack of productive MERS-CoV infection. Surprisingly, our findings for TOMF activity in MERS-CoV-infected MDMs and immature MDDCs differed from results of previous studies in Vero E6 cells. TOMF showed minimal activity against MERS-CoV infection in MDMs. TOMF also had little antiviral activity in immature MDDCs until reaching a high concentration (>20 μM) that was associated with high cytotoxicity. TOMF is an estrogen receptor modulator that has been shown to inhibit filoviruses [38] and block both MERS-CoV and SARS-CoV with EC_50_s12.9 and 11.97μM, respectively, in Vero E6 cells [13]. This drug inhibits late phrase of virus entry by interfering the fusion of filoviruses [38]. However, in primary cells, the EC_50_ increased to 38 μM though lower cytotoxicity was seen (CC_5 0_ = 22.54 μM). Thus, the therapeutic window for effective treatment with TOMF in primary APCs is narrow.

Previously, CQ, a well-established antiparasitic agent, showed strong inhibition on MERS-CoV and SARS-CoV with low EC_50_s (range from 5.76 to 12.9 μM) and low toxicity [13]. CQ likely accumulates in lysosomes where it sequesters protons and increases the pH. The drug interacts with a variety of host proteins and cellular processes, resulting in modulation of immune responses [39]. CQ has also been reported to inhibit replication of multiple viruses such as flaviviruses [40], influenza viruses [41, 42], HIV [40], Ebola [43] and Nipah viruses [44] *in vitro*. In our study, CQ had minimal anti-MERS activity in either MERS-CoV-infected MDMs or immature MDDCs, though appreciable cytotoxicity was not observed.

CPZ is a neurotransmitter inhibitor, which was the first antipsychotic drug developed for treatment of schizophrenia treatment [45]. CPZ inhibits clathrin-mediated endocytosis through preventing the formulation of clathrin-coated pits at the plasma membrane [46]. Previous work has reported that CPZ inhibits MERS-CoV infection of Huh 7 cells with an EC_50_ of 4.9, vs CC_50_ of 21.3 μM [14]. In our primary cells, some activity against MERS-CoV was observed in CPZ-pretreated MDMs and immature MDDCs (EC_50_ = 13.58 μM). However, the high cytotoxicity (25.64 μM) observed with CPZ on both types of primary cells precludes further development. These studies demonstrate the tested compounds, each of which were shown to be efficacious in continuous cell lines, were not effective at inhibiting MERS-CoV infection in primary APCs.

In conclusion, MERS-CoV infection in primary MDMs induces overexpression of inflammatory cytokines/chemokines potentially leading to tissue damage and systemic virus dissemination. On the other hand, the mechanism of immune escape through MDDC results in inefficient viral clearance, which may explain the high pathogenicity and clinical manifestations seen in MERS. This study also provides the first demonstration of medical countermeasure evaluation for MERS-CoV in primary APCs and illustrates the importance of using non-immortalized primary cells for the evaluation of drug efficacy. These data also suggest that, given the established mechanisms of action of TOMF, CQ and CPZ, the mechanisms of virus internalization and exocytosis may be different from those used in established cell lines. Before performing *in vivo* studies, potential compounds should be evaluated in primary cells as major differences may occur between immortalized cell lines and primary cells.
